# *catena*-Poly[[(tri­phenyl­phosphane-κ*P*)silver(I)]-μ-4,4′-bi­pyridine-κ^2^ *N*:*N*′-[(tri­phenyl­phosphane-κ*P*)silver(I)]-di-μ-chlorido]

**DOI:** 10.1107/S160053681301413X

**Published:** 2013-05-25

**Authors:** Xiao-Ming Song, Feng Hu, Hua-Tian Shi, Qun Chen, Qian-Feng Zhang

**Affiliations:** a Department of Applied Chemistry, School of Petrochemical Engineering, Changzhou University, Jiangsu 213164, People’s Republic of China; b Institute of Molecular Engineering and Applied Chemistry, Anhui University of Technology, Ma’anshan, Anhui 243002, People’s Republic of China

## Abstract

In the title coordination polymer, [Ag_2_Cl_2_(C_10_H_8_N_2_)(C_18_H_15_P)_2_]_*n*_, the Ag^I^ cation is coordinated by a 4,4′-bi­pyridine N atom, a tri­phenyl­phosphane P atom and two Cl^−^ anions in a distorted tetra­hedral geometry. The 4,4-bi­pyridine and Cl^−^ anions bridge the Ag^I^ cations, forming polymeric chains running along [21-1]. In the crystal, weak C—H⋯Cl inter­actions link the polymeric chains into a three-dimensiona supra­molecular architecture.

## Related literature

For background to silver coordination polymers, see: Hung-Low & Klausmeyer (2008 ▶); Mishra *et al.* (2007 ▶); Pyykkö (2004 ▶); Yam & Lo (1999 ▶); Zaworotko (1994 ▶). For related structures, see: Lu *et al.* (1997 ▶); Sampanthar & Vittal (2000 ▶); Sun *et al.* (2009 ▶).
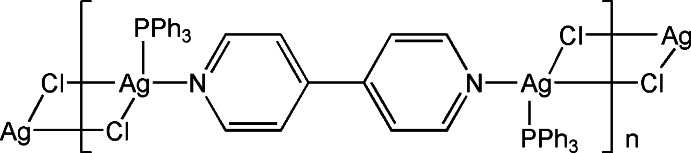


## Experimental

### 

#### Crystal data

[Ag_2_Cl_2_(C_10_H_8_N_2_)(C_18_H_15_P)_2_]
*M* *_r_* = 967.36
Triclinic, 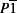

*a* = 9.1042 (16) Å
*b* = 13.887 (2) Å
*c* = 17.826 (3) Å
α = 70.753 (3)°
β = 79.332 (4)°
γ = 75.190 (3)°
*V* = 2044.5 (6) Å^3^
*Z* = 2
Mo *K*α radiation
μ = 1.20 mm^−1^
*T* = 296 K
0.23 × 0.17 × 0.14 mm

#### Data collection

Bruker SMART APEXII CCD area-detector diffractometer
Absorption correction: multi-scan (*SADABS*; Bruker, 2001 ▶) *T*_min_ = 0.770, *T*_max_ = 0.850
14039 measured reflections
9594 independent reflections
6338 reflections with *I* > 2σ(*I*)
*R*_int_ = 0.018

#### Refinement

*R*[*F* ^2^ > 2σ(*F* ^2^)] = 0.039
*wR*(*F* ^2^) = 0.092
*S* = 1.01
9594 reflections
487 parameters
H-atom parameters constrained
Δρ_max_ = 0.59 e Å^−3^
Δρ_min_ = −0.44 e Å^−3^

### 

Data collection: *APEX2* (Bruker, 2007 ▶); cell refinement: *SAINT* (Bruker, 2007 ▶); data reduction: *SAINT*; program(s) used to solve structure: *SHELXTL* (Sheldrick, 2008 ▶); program(s) used to refine structure: *SHELXTL*; molecular graphics: *SHELXTL*; software used to prepare material for publication: *SHELXTL*.

## Supplementary Material

Crystal structure: contains datablock(s) I, global. DOI: 10.1107/S160053681301413X/xu5704sup1.cif

Structure factors: contains datablock(s) I. DOI: 10.1107/S160053681301413X/xu5704Isup2.hkl

Additional supplementary materials:  crystallographic information; 3D view; checkCIF report

## Figures and Tables

**Table 1:** Selected bond lengths (Å)

Ag1—P1	2.4069 (9)
Ag1—N1	2.430 (3)
Ag1—Cl1	2.5709 (10)
Ag1—Cl1^i^	2.6639 (10)
Ag2—P2	2.4162 (9)
Ag2—N2	2.386 (3)
Ag2—Cl2	2.6111 (9)
Ag2—Cl2^ii^	2.6809 (10)

Symmetry codes: (i) 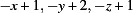; (ii) 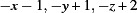.

**Table 2:** Hydrogen-bond geometry (Å, °)

*D*—H⋯*A*	*D*—H	H⋯*A*	*D*⋯*A*	*D*—H⋯*A*
C9—H9⋯Cl2^iii^	0.93	2.82	3.669 (4)	153

Symmetry code: (iii) 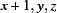.

**Fig. 1.**
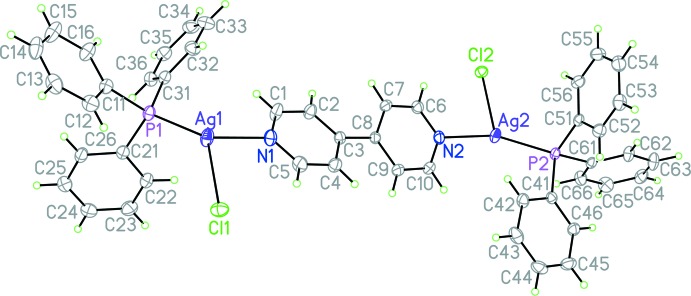
The structure of the title coordination polymer, showing the atom-numbering scheme and displacement ellipsoids at the 50% probability level.

**Fig. 2.**
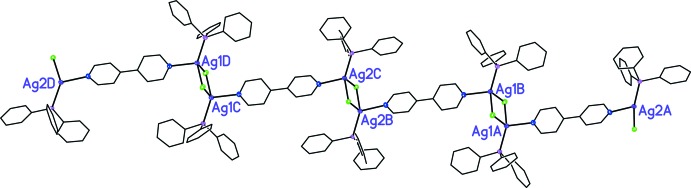
A view the [(µ-4,4'-bipy)(µ-Cl)2(AgPPh3)2]n chain, four unit cells along as drawn by ORTEP with 50% probability level.

## supplementary crystallographic information

### Comment

There has been an extensive interest in d^10^ metal complexes with phosphane ligands due to their potential application in luminescence (Yam & Lo, 1999), for this important reason, the study of d^10^ "closed-shell" interactions that exist between the monovalent elements of group 11 has been active for many years (Pyykko, 2004). Actually, these metal-metal interactions are typically associated with the ligand-bridged, hydrogen-bonded and pi-pi stacked effects, which may result in formation of supramolecular assemblies (Mishra *et al.*, 2007; Zaworotko, 1994). Metal coordination polymers with linear spacer ligands have been exploited by many research workers to construct a variety of network structures. Specifically silver(I) ion has been extensively used in inorganic crystal engineering using self-assembly of tailored building-blocks (Hung-Low & Klausmeyer, 2008). In recent decade, self-assembly of silver(I) salts with different aliphatic dinitrile ligands such as 4,4'-bipyridyl (4,4'-bpy) have also been successfully made resulting into novel coordination polymers (Sampanthar & Vittal, 2000). With this in mind, we have chosen a simple AgCl salt and a linear spacer 4,4'-bpy and allowed them to react separately with PPh_3_ as an ancillary ligand. The results of this work are reported in this paper.

The title coordination polymer crystallizes in the triclinic centrosymmetric *P*-1 space group with *Z* = 2 as it contains one half molecule in an asymmetric unit. A view of the structure of building block in the title polymeric complex is depicted in Fig. 1. The structure consists of {(µ-Cl)(AgPPh_3_)}_2_ units bridged by 4,4'-bipy ligands to form a zig-zig infinite chain, as shown in Fig. 2. This structure is isostructural to [(µ-4,4'-bipy)(µ-I)_2_(AgPPh_3_)_2_]_n_ (Sampanthar & Vittal, 2000) and [(µ-4,4'-bipy)(µ-Cl)_2_(CuPPh_3_)_2_]_n_ (Lu *et al.*, 1997). The coordination polymer possesses to the crystallographic inversion center through the middle of Ag_2_Cl_2_ squares. Two pyridine rings in the 4,4'-bipy are non-planar with dihedral angle of 22.4 (3)°. The average Ag···Ag distance in the Ag_2_Cl_2_ ring is 3.392 (1) Å, which is slightly longer than that of 3.139 (1) Å in [(µ-4,4'-bipy)(µ-I)_2_(AgPPh_3_)_2_]_n_ (Sampanthar & Vittal, 2000). Each silver(I) ion in the title coordination polymer is coordinated by one nitrogen atom of 4,4'-bipy ligand, one phosphorous atom of PPh_3_ ligand and two chloride atoms, leading to the distorted tetrahedron with the angles around silver varying from 94.24 (7)° to 130.08 (3)°. Two silver(I) ions are separated by 4,4'-bipy groups at a distances of 10.957 (1) Å along with axial direction. The average Ag—N and Ag—P bond lengths are 2.408 (3) Å and 2.4116 (9) Å, respectively, which almost similar to the values reported in the related other complexes (Sampanthar & Vittal, 2000, Sun *et al.*, 2009). The Ag—Cl—Ag angles of 97.37 (3)° and 102.05 (2)° in the title coordination polymer are obviously larger than the Ag—I—Ag angle of 66.27 (1)° in [(µ-4,4'-bipy)(µ-I)2(AgPPh_3_)_2_]_n_ (Sampanthar & Vittal, 2000).

### Experimental

AgCl (0.080 g, 0.56 mmol) and PPh_3_ (0.162 g, 0.62 mmol) were mixed together and stirred in a mixture of CH_2_Cl_2_ (15 mL) and MeCN (5 mL) for 45 min to get a clear solution. An MeCN solution (5 mL) of 4,4'-bipyridyl (0.043 g, 0.28 mmol) was added slowly to the above solution with stirring. The clear solution was filtered and left for slow evaporation. Colorless crystals were collected by decanting the solvent and washed with MeOH (5 mL) and Et_2_O (5 x 2 mL) then air-dried. Yield: 157 mg, 58 %. Analysis for C_46_H_38_N_2_Cl_2_P_2_Ag_2_: calcd C 57.11, H 3.96, N 2.90 %; found C 57.07, H 4.03, N 2.88 %.

### Refinement

H atoms were placed in geometrically idealized positions and refined in riding model with C—H = 0.93 Å and *U*_iso_(H) = 1.2*U*_eq_(C)].

### Crystal data

**Table:**

[Ag_2_Cl_2_(C_10_H_8_N_2_)(C_18_H_15_P)_2_]	*Z* = 2
*M**_r_* = 967.36	*F*(000) = 972
Triclinic, *P*1	*D*_x_ = 1.571 Mg m^−^^3^
Hall symbol: -P 1	Mo *K*α radiation, λ = 0.71073 Å
*a* = 9.1042 (16) Å	Cell parameters from 4454 reflections
*b* = 13.887 (2) Å	θ = 2.3–28.9°
*c* = 17.826 (3) Å	µ = 1.20 mm^−^^1^
α = 70.753 (3)°	*T* = 296 K
β = 79.332 (4)°	Block, light yellow
γ = 75.190 (3)°	0.23 × 0.17 × 0.14 mm
*V* = 2044.5 (6) Å^3^	

### Data collection

**Table:**

Bruker SMART APEXII CCD area-detector diffractometer	9594 independent reflections
Radiation source: fine-focus sealed tube	6338 reflections with *I* > 2σ(*I*)
Graphite monochromator	*R*_int_ = 0.018
φ and ω scans	θ_max_ = 29.2°, θ_min_ = 1.6°
Absorption correction: multi-scan (*SADABS*; Bruker, 2001)	*h* = −10→12
*T*_min_ = 0.770, *T*_max_ = 0.850	*k* = −12→19
14039 measured reflections	*l* = −15→23

### Refinement

**Table:**

Refinement on *F*^2^	Primary atom site location: structure-invariant direct methods
Least-squares matrix: full	Secondary atom site location: difference Fourier map
*R*[*F*^2^ > 2σ(*F*^2^)] = 0.039	Hydrogen site location: inferred from neighbouring sites
*wR*(*F*^2^) = 0.092	H-atom parameters constrained
*S* = 1.01	*w* = 1/[σ^2^(*F*_o_^2^) + (0.0251*P*)^2^ + 1.3705*P*] where *P* = (*F*_o_^2^ + 2*F*_c_^2^)/3
9594 reflections	(Δ/σ)_max_ = 0.001
487 parameters	Δρ_max_ = 0.59 e Å^−^^3^
0 restraints	Δρ_min_ = −0.44 e Å^−^^3^

### Special details

**Table:**

Geometry. All esds (except the esd in the dihedral angle between two l.s. planes) are estimated using the full covariance matrix. The cell esds are taken into account individually in the estimation of esds in distances, angles and torsion angles; correlations between esds in cell parameters are only used when they are defined by crystal symmetry. An approximate (isotropic) treatment of cell esds is used for estimating esds involving l.s. planes.
Refinement. Refinement of F^2^ against ALL reflections. The weighted R-factor wR and goodness of fit S are based on F^2^, conventional R-factors R are based on F, with F set to zero for negative F^2^. The threshold expression of F^2^ > 2sigma(F^2^) is used only for calculating R-factors(gt) etc. and is not relevant to the choice of reflections for refinement. R-factors based on F^2^ are statistically about twice as large as those based on F, and R- factors based on ALL data will be even larger.

### Fractional atomic coordinates and isotropic or equivalent isotropic displacement parameters (Å^2^)

**Table:**

	*x*	*y*	*z*	*U*_iso_*/*U*_eq_	
Ag1	0.34094 (3)	1.07363 (2)	0.539814 (18)	0.05839 (9)	
Ag2	−0.34478 (3)	0.431910 (18)	0.960631 (16)	0.05064 (8)	
Cl1	0.59763 (10)	0.97839 (8)	0.59551 (5)	0.0607 (2)	
Cl2	−0.60355 (9)	0.52295 (6)	0.90046 (5)	0.04829 (19)	
P1	0.20766 (10)	1.25225 (6)	0.52007 (5)	0.0454 (2)	
P2	−0.21810 (8)	0.25279 (6)	0.97259 (5)	0.03729 (17)	
N1	0.1967 (3)	0.9471 (2)	0.62893 (17)	0.0524 (7)	
N2	−0.2206 (3)	0.5717 (2)	0.88543 (16)	0.0469 (6)	
C1	0.0440 (4)	0.9661 (3)	0.6366 (2)	0.0638 (10)	
H1	−0.0076	1.0305	0.6071	0.077*	
C2	−0.0412 (4)	0.8954 (3)	0.6859 (2)	0.0558 (9)	
H2	−0.1473	0.9127	0.6886	0.067*	
C3	0.0305 (3)	0.7994 (2)	0.73101 (18)	0.0416 (7)	
C4	0.1897 (4)	0.7796 (3)	0.7233 (2)	0.0559 (9)	
H4	0.2444	0.7161	0.7524	0.067*	
C5	0.2660 (4)	0.8549 (3)	0.6722 (2)	0.0608 (10)	
H5	0.3723	0.8397	0.6680	0.073*	
C6	−0.2807 (4)	0.6486 (3)	0.8242 (2)	0.0490 (8)	
H6	−0.3798	0.6521	0.8155	0.059*	
C7	−0.2039 (4)	0.7233 (2)	0.77318 (19)	0.0468 (8)	
H7	−0.2510	0.7751	0.7313	0.056*	
C8	−0.0565 (3)	0.7208 (2)	0.78454 (18)	0.0391 (7)	
C9	0.0055 (4)	0.6417 (3)	0.8484 (2)	0.0530 (9)	
H9	0.1040	0.6365	0.8589	0.064*	
C10	−0.0804 (4)	0.5706 (3)	0.8963 (2)	0.0555 (9)	
H10	−0.0366	0.5184	0.9390	0.067*	
C11	0.2506 (4)	1.3467 (3)	0.42354 (19)	0.0480 (8)	
C12	0.4036 (4)	1.3413 (3)	0.3919 (2)	0.0631 (10)	
H12	0.4798	1.2891	0.4181	0.076*	
C13	0.4405 (5)	1.4153 (4)	0.3205 (2)	0.0745 (12)	
H13	0.5423	1.4133	0.2995	0.089*	
C14	0.3284 (7)	1.4907 (3)	0.2812 (3)	0.0820 (14)	
H14	0.3544	1.5396	0.2336	0.098*	
C15	0.1790 (6)	1.4946 (3)	0.3112 (2)	0.0799 (13)	
H15	0.1029	1.5451	0.2835	0.096*	
C16	0.1408 (5)	1.4234 (3)	0.3828 (2)	0.0637 (10)	
H16	0.0387	1.4275	0.4037	0.076*	
C21	0.2366 (3)	1.3116 (3)	0.5928 (2)	0.0460 (7)	
C22	0.2340 (5)	1.2515 (3)	0.6731 (2)	0.0629 (10)	
H22	0.2216	1.1830	0.6877	0.075*	
C23	0.2498 (5)	1.2930 (4)	0.7306 (2)	0.0761 (12)	
H23	0.2447	1.2530	0.7840	0.091*	
C24	0.2731 (5)	1.3926 (4)	0.7100 (3)	0.0721 (11)	
H24	0.2849	1.4200	0.7491	0.087*	
C25	0.2789 (5)	1.4516 (3)	0.6310 (3)	0.0729 (11)	
H25	0.2951	1.5191	0.6165	0.087*	
C26	0.2608 (4)	1.4113 (3)	0.5732 (2)	0.0577 (9)	
H26	0.2649	1.4520	0.5200	0.069*	
C31	0.0001 (4)	1.2669 (2)	0.52947 (19)	0.0445 (7)	
C32	−0.0577 (4)	1.2105 (3)	0.4944 (2)	0.0605 (9)	
H32	0.0088	1.1696	0.4651	0.073*	
C33	−0.2136 (5)	1.2147 (4)	0.5027 (3)	0.0779 (12)	
H33	−0.2515	1.1756	0.4803	0.093*	
C34	−0.3120 (5)	1.2778 (4)	0.5448 (2)	0.0697 (11)	
H34	−0.4167	1.2810	0.5507	0.084*	
C35	−0.2569 (4)	1.3350 (3)	0.5774 (2)	0.0602 (9)	
H35	−0.3240	1.3783	0.6046	0.072*	
C36	−0.1008 (4)	1.3293 (3)	0.5703 (2)	0.0510 (8)	
H36	−0.0640	1.3682	0.5935	0.061*	
C41	−0.0208 (3)	0.2281 (2)	0.99274 (19)	0.0405 (7)	
C42	0.0690 (4)	0.2968 (3)	0.9441 (2)	0.0515 (8)	
H42	0.0294	0.3501	0.9007	0.062*	
C43	0.2169 (4)	0.2874 (3)	0.9591 (3)	0.0659 (11)	
H43	0.2756	0.3344	0.9262	0.079*	
C44	0.2764 (4)	0.2080 (3)	1.0228 (3)	0.0658 (11)	
H44	0.3754	0.2015	1.0334	0.079*	
C45	0.1892 (4)	0.1383 (3)	1.0709 (2)	0.0639 (10)	
H45	0.2300	0.0839	1.1134	0.077*	
C46	0.0410 (4)	0.1492 (3)	1.0561 (2)	0.0509 (8)	
H46	−0.0177	0.1025	1.0894	0.061*	
C51	−0.2043 (3)	0.2094 (2)	0.88489 (18)	0.0390 (7)	
C52	−0.0742 (4)	0.1461 (3)	0.8581 (2)	0.0512 (8)	
H52	0.0140	0.1270	0.8834	0.061*	
C53	−0.0759 (4)	0.1116 (3)	0.7940 (2)	0.0590 (9)	
H53	0.0108	0.0685	0.7769	0.071*	
C54	−0.2042 (5)	0.1402 (3)	0.7555 (2)	0.0654 (10)	
H54	−0.2037	0.1174	0.7119	0.078*	
C55	−0.3335 (5)	0.2025 (3)	0.7812 (2)	0.0658 (10)	
H55	−0.4212	0.2207	0.7556	0.079*	
C56	−0.3337 (4)	0.2382 (3)	0.8448 (2)	0.0524 (8)	
H56	−0.4209	0.2817	0.8611	0.063*	
C61	−0.3002 (3)	0.1526 (2)	1.05223 (18)	0.0392 (7)	
C62	−0.3049 (4)	0.0577 (3)	1.0440 (2)	0.0542 (9)	
H62	−0.2648	0.0424	0.9963	0.065*	
C63	−0.3694 (5)	−0.0147 (3)	1.1068 (2)	0.0696 (11)	
H63	−0.3700	−0.0789	1.1015	0.084*	
C64	−0.4319 (4)	0.0076 (3)	1.1765 (2)	0.0632 (10)	
H64	−0.4768	−0.0406	1.2180	0.076*	
C65	−0.4280 (5)	0.1006 (3)	1.1847 (2)	0.0688 (11)	
H65	−0.4692	0.1153	1.2325	0.083*	
C66	−0.3637 (4)	0.1737 (3)	1.1232 (2)	0.0551 (9)	
H66	−0.3631	0.2374	1.1296	0.066*	

### Atomic displacement parameters (Å^2^)

**Table:**

	*U*^11^	*U*^22^	*U*^33^	*U*^12^	*U*^13^	*U*^23^
Ag1	0.04979 (16)	0.03807 (14)	0.0748 (2)	−0.00268 (11)	0.00765 (13)	−0.01311 (13)
Ag2	0.04678 (15)	0.03340 (13)	0.06182 (17)	−0.00289 (10)	0.00090 (12)	−0.00901 (11)
Cl1	0.0483 (5)	0.0784 (6)	0.0510 (5)	−0.0104 (4)	−0.0062 (4)	−0.0153 (4)
Cl2	0.0403 (4)	0.0520 (5)	0.0485 (5)	−0.0089 (3)	−0.0058 (3)	−0.0095 (4)
P1	0.0429 (4)	0.0348 (4)	0.0514 (5)	−0.0040 (3)	0.0009 (4)	−0.0100 (4)
P2	0.0322 (4)	0.0296 (4)	0.0454 (5)	−0.0046 (3)	−0.0019 (3)	−0.0078 (3)
N1	0.0519 (17)	0.0442 (16)	0.0566 (18)	−0.0153 (13)	0.0060 (14)	−0.0115 (13)
N2	0.0563 (17)	0.0388 (14)	0.0472 (16)	−0.0181 (12)	−0.0025 (13)	−0.0104 (12)
C1	0.053 (2)	0.0410 (19)	0.077 (3)	−0.0055 (16)	0.0042 (19)	0.0007 (17)
C2	0.0427 (18)	0.0443 (19)	0.068 (2)	−0.0069 (15)	−0.0014 (16)	−0.0042 (16)
C3	0.0412 (16)	0.0386 (16)	0.0449 (18)	−0.0114 (13)	−0.0023 (14)	−0.0113 (13)
C4	0.0414 (18)	0.049 (2)	0.068 (2)	−0.0087 (15)	−0.0081 (17)	−0.0046 (17)
C5	0.0434 (19)	0.067 (2)	0.070 (2)	−0.0206 (18)	0.0038 (17)	−0.016 (2)
C6	0.0452 (18)	0.0458 (18)	0.056 (2)	−0.0146 (15)	−0.0048 (16)	−0.0110 (16)
C7	0.0448 (18)	0.0420 (17)	0.0492 (19)	−0.0092 (14)	−0.0151 (15)	−0.0024 (14)
C8	0.0414 (16)	0.0339 (15)	0.0425 (17)	−0.0083 (12)	−0.0048 (13)	−0.0115 (13)
C9	0.0489 (19)	0.0449 (19)	0.062 (2)	−0.0109 (15)	−0.0210 (17)	−0.0037 (16)
C10	0.068 (2)	0.0412 (18)	0.053 (2)	−0.0122 (17)	−0.0211 (18)	0.0005 (15)
C11	0.0536 (19)	0.0456 (18)	0.0419 (18)	−0.0091 (15)	0.0022 (15)	−0.0141 (14)
C12	0.058 (2)	0.078 (3)	0.055 (2)	−0.020 (2)	0.0035 (18)	−0.022 (2)
C13	0.079 (3)	0.093 (3)	0.063 (3)	−0.045 (3)	0.022 (2)	−0.034 (2)
C14	0.132 (4)	0.056 (3)	0.056 (3)	−0.036 (3)	0.020 (3)	−0.018 (2)
C15	0.111 (4)	0.051 (2)	0.053 (2)	0.002 (2)	0.008 (2)	−0.0067 (18)
C16	0.071 (2)	0.049 (2)	0.053 (2)	0.0042 (18)	0.0074 (18)	−0.0124 (17)
C21	0.0366 (16)	0.0446 (18)	0.052 (2)	−0.0065 (13)	−0.0017 (14)	−0.0108 (15)
C22	0.076 (3)	0.054 (2)	0.053 (2)	−0.0228 (19)	−0.0103 (19)	0.0001 (18)
C23	0.091 (3)	0.086 (3)	0.048 (2)	−0.024 (3)	−0.014 (2)	−0.007 (2)
C24	0.080 (3)	0.080 (3)	0.066 (3)	−0.019 (2)	−0.020 (2)	−0.026 (2)
C25	0.086 (3)	0.060 (2)	0.081 (3)	−0.020 (2)	−0.022 (2)	−0.020 (2)
C26	0.068 (2)	0.0422 (19)	0.057 (2)	−0.0104 (17)	−0.0109 (18)	−0.0065 (16)
C31	0.0426 (17)	0.0395 (17)	0.0458 (18)	−0.0078 (13)	−0.0025 (14)	−0.0072 (14)
C32	0.064 (2)	0.065 (2)	0.057 (2)	−0.0107 (19)	−0.0088 (19)	−0.0252 (19)
C33	0.075 (3)	0.089 (3)	0.081 (3)	−0.021 (3)	−0.033 (2)	−0.024 (3)
C34	0.049 (2)	0.088 (3)	0.065 (3)	−0.017 (2)	−0.0140 (19)	−0.007 (2)
C35	0.0456 (19)	0.065 (2)	0.059 (2)	−0.0056 (17)	−0.0021 (17)	−0.0114 (18)
C36	0.0433 (18)	0.0493 (19)	0.059 (2)	−0.0103 (15)	0.0003 (15)	−0.0168 (16)
C41	0.0366 (15)	0.0364 (15)	0.0501 (19)	−0.0047 (12)	−0.0034 (13)	−0.0181 (14)
C42	0.0432 (18)	0.0450 (18)	0.064 (2)	−0.0134 (14)	0.0007 (16)	−0.0131 (16)
C43	0.048 (2)	0.071 (3)	0.089 (3)	−0.0266 (19)	0.009 (2)	−0.035 (2)
C44	0.0371 (18)	0.085 (3)	0.089 (3)	−0.0070 (19)	−0.0128 (19)	−0.046 (3)
C45	0.050 (2)	0.068 (3)	0.070 (3)	0.0022 (18)	−0.0215 (19)	−0.019 (2)
C46	0.0411 (17)	0.0444 (18)	0.065 (2)	−0.0060 (14)	−0.0097 (16)	−0.0128 (16)
C51	0.0378 (15)	0.0311 (14)	0.0425 (17)	−0.0092 (12)	−0.0017 (13)	−0.0035 (12)
C52	0.0418 (17)	0.051 (2)	0.057 (2)	−0.0059 (15)	−0.0044 (15)	−0.0155 (16)
C53	0.067 (2)	0.052 (2)	0.056 (2)	−0.0120 (18)	0.0065 (19)	−0.0201 (17)
C54	0.092 (3)	0.060 (2)	0.047 (2)	−0.024 (2)	−0.004 (2)	−0.0149 (18)
C55	0.069 (3)	0.068 (3)	0.061 (2)	−0.018 (2)	−0.024 (2)	−0.008 (2)
C56	0.0443 (18)	0.052 (2)	0.053 (2)	−0.0082 (15)	−0.0091 (16)	−0.0051 (16)
C61	0.0295 (14)	0.0334 (15)	0.0488 (18)	−0.0060 (11)	−0.0051 (13)	−0.0046 (13)
C62	0.063 (2)	0.0381 (17)	0.055 (2)	−0.0130 (15)	0.0066 (17)	−0.0100 (15)
C63	0.081 (3)	0.0387 (19)	0.079 (3)	−0.0216 (19)	0.008 (2)	−0.0071 (18)
C64	0.058 (2)	0.054 (2)	0.061 (2)	−0.0197 (18)	−0.0008 (18)	0.0072 (18)
C65	0.072 (3)	0.083 (3)	0.049 (2)	−0.030 (2)	0.0130 (19)	−0.017 (2)
C66	0.061 (2)	0.054 (2)	0.052 (2)	−0.0216 (17)	0.0071 (17)	−0.0181 (16)

### Geometric parameters (Å, º)

**Table:**

Ag1—P1	2.4069 (9)	C22—H22	0.9300
Ag1—N1	2.430 (3)	C23—C24	1.371 (6)
Ag1—Cl1	2.5709 (10)	C23—H23	0.9300
Ag1—Cl1^i^	2.6639 (10)	C24—C25	1.376 (6)
Ag2—P2	2.4162 (9)	C24—H24	0.9300
Ag2—N2	2.386 (3)	C25—C26	1.376 (5)
Ag2—Cl2	2.6111 (9)	C25—H25	0.9300
Ag2—Cl2^ii^	2.6809 (10)	C26—H26	0.9300
Ag2—Ag2^ii^	3.3292 (6)	C31—C36	1.373 (4)
Cl1—Ag1^i^	2.6639 (11)	C31—C32	1.392 (5)
Cl2—Ag2^ii^	2.6809 (10)	C32—C33	1.387 (5)
P1—C31	1.830 (3)	C32—H32	0.9300
P1—C11	1.833 (3)	C33—C34	1.384 (6)
P1—C21	1.836 (4)	C33—H33	0.9300
P2—C41	1.821 (3)	C34—C35	1.355 (6)
P2—C51	1.825 (3)	C34—H34	0.9300
P2—C61	1.829 (3)	C35—C36	1.388 (5)
N1—C5	1.322 (5)	C35—H35	0.9300
N1—C1	1.336 (4)	C36—H36	0.9300
N2—C10	1.321 (4)	C41—C46	1.378 (4)
N2—C6	1.336 (4)	C41—C42	1.387 (4)
C1—C2	1.381 (5)	C42—C43	1.386 (5)
C1—H1	0.9300	C42—H42	0.9300
C2—C3	1.375 (4)	C43—C44	1.378 (6)
C2—H2	0.9300	C43—H43	0.9300
C3—C4	1.394 (4)	C44—C45	1.376 (5)
C3—C8	1.485 (4)	C44—H44	0.9300
C4—C5	1.384 (5)	C45—C46	1.384 (5)
C4—H4	0.9300	C45—H45	0.9300
C5—H5	0.9300	C46—H46	0.9300
C6—C7	1.381 (4)	C51—C56	1.393 (4)
C6—H6	0.9300	C51—C52	1.394 (4)
C7—C8	1.383 (4)	C52—C53	1.380 (5)
C7—H7	0.9300	C52—H52	0.9300
C8—C9	1.385 (4)	C53—C54	1.368 (5)
C9—C10	1.380 (5)	C53—H53	0.9300
C9—H9	0.9300	C54—C55	1.374 (6)
C10—H10	0.9300	C54—H54	0.9300
C11—C16	1.370 (5)	C55—C56	1.379 (5)
C11—C12	1.395 (5)	C55—H55	0.9300
C12—C13	1.396 (5)	C56—H56	0.9300
C12—H12	0.9300	C61—C66	1.382 (4)
C13—C14	1.364 (6)	C61—C62	1.385 (4)
C13—H13	0.9300	C62—C63	1.388 (5)
C14—C15	1.361 (6)	C62—H62	0.9300
C14—H14	0.9300	C63—C64	1.364 (5)
C15—C16	1.382 (5)	C63—H63	0.9300
C15—H15	0.9300	C64—C65	1.357 (6)
C16—H16	0.9300	C64—H64	0.9300
C21—C26	1.377 (5)	C65—C66	1.381 (5)
C21—C22	1.399 (5)	C65—H65	0.9300
C22—C23	1.374 (6)	C66—H66	0.9300
			
P1—Ag1—N1	114.03 (7)	C23—C22—C21	120.4 (4)
P1—Ag1—Cl1	130.08 (3)	C23—C22—H22	119.8
N1—Ag1—Cl1	95.47 (8)	C21—C22—H22	119.8
P1—Ag1—Cl1^i^	112.23 (3)	C24—C23—C22	120.7 (4)
N1—Ag1—Cl1^i^	103.72 (7)	C24—C23—H23	119.7
Cl1—Ag1—Cl1^i^	97.37 (3)	C22—C23—H23	119.7
N2—Ag2—P2	120.95 (7)	C23—C24—C25	119.3 (4)
N2—Ag2—Cl2	94.24 (7)	C23—C24—H24	120.3
P2—Ag2—Cl2	124.50 (3)	C25—C24—H24	120.3
N2—Ag2—Cl2^ii^	96.68 (7)	C26—C25—C24	120.4 (4)
P2—Ag2—Cl2^ii^	113.34 (3)	C26—C25—H25	119.8
Cl2—Ag2—Cl2^ii^	102.05 (2)	C24—C25—H25	119.8
N2—Ag2—Ag2^ii^	98.72 (7)	C25—C26—C21	121.1 (4)
P2—Ag2—Ag2^ii^	139.76 (2)	C25—C26—H26	119.5
Cl2—Ag2—Ag2^ii^	51.96 (2)	C21—C26—H26	119.5
Cl2^ii^—Ag2—Ag2^ii^	50.090 (19)	C36—C31—C32	118.6 (3)
Ag1—Cl1—Ag1^i^	82.63 (3)	C36—C31—P1	123.4 (3)
Ag2—Cl2—Ag2^ii^	77.95 (2)	C32—C31—P1	118.1 (3)
C31—P1—C11	103.84 (15)	C33—C32—C31	120.7 (4)
C31—P1—C21	103.95 (15)	C33—C32—H32	119.7
C11—P1—C21	103.26 (15)	C31—C32—H32	119.7
C31—P1—Ag1	112.39 (11)	C34—C33—C32	119.3 (4)
C11—P1—Ag1	117.37 (11)	C34—C33—H33	120.4
C21—P1—Ag1	114.55 (11)	C32—C33—H33	120.4
C41—P2—C51	104.34 (14)	C35—C34—C33	120.4 (4)
C41—P2—C61	104.46 (14)	C35—C34—H34	119.8
C51—P2—C61	102.45 (14)	C33—C34—H34	119.8
C41—P2—Ag2	111.14 (10)	C34—C35—C36	120.3 (4)
C51—P2—Ag2	116.42 (10)	C34—C35—H35	119.8
C61—P2—Ag2	116.60 (10)	C36—C35—H35	119.8
C5—N1—C1	116.4 (3)	C31—C36—C35	120.7 (3)
C5—N1—Ag1	121.4 (2)	C31—C36—H36	119.6
C1—N1—Ag1	122.2 (2)	C35—C36—H36	119.6
C10—N2—C6	116.2 (3)	C46—C41—C42	118.4 (3)
C10—N2—Ag2	121.3 (2)	C46—C41—P2	123.8 (2)
C6—N2—Ag2	122.1 (2)	C42—C41—P2	117.7 (2)
N1—C1—C2	123.6 (3)	C43—C42—C41	121.0 (3)
N1—C1—H1	118.2	C43—C42—H42	119.5
C2—C1—H1	118.2	C41—C42—H42	119.5
C3—C2—C1	120.1 (3)	C44—C43—C42	119.6 (4)
C3—C2—H2	119.9	C44—C43—H43	120.2
C1—C2—H2	119.9	C42—C43—H43	120.2
C2—C3—C4	116.3 (3)	C45—C44—C43	119.9 (3)
C2—C3—C8	122.0 (3)	C45—C44—H44	120.0
C4—C3—C8	121.7 (3)	C43—C44—H44	120.0
C5—C4—C3	119.7 (3)	C44—C45—C46	120.0 (4)
C5—C4—H4	120.1	C44—C45—H45	120.0
C3—C4—H4	120.1	C46—C45—H45	120.0
N1—C5—C4	123.8 (3)	C41—C46—C45	121.0 (3)
N1—C5—H5	118.1	C41—C46—H46	119.5
C4—C5—H5	118.1	C45—C46—H46	119.5
N2—C6—C7	123.6 (3)	C56—C51—C52	118.6 (3)
N2—C6—H6	118.2	C56—C51—P2	117.7 (2)
C7—C6—H6	118.2	C52—C51—P2	123.7 (2)
C6—C7—C8	119.7 (3)	C53—C52—C51	120.1 (3)
C6—C7—H7	120.1	C53—C52—H52	120.0
C8—C7—H7	120.1	C51—C52—H52	120.0
C7—C8—C9	116.7 (3)	C54—C53—C52	120.6 (4)
C7—C8—C3	121.6 (3)	C54—C53—H53	119.7
C9—C8—C3	121.7 (3)	C52—C53—H53	119.7
C10—C9—C8	119.4 (3)	C53—C54—C55	120.1 (4)
C10—C9—H9	120.3	C53—C54—H54	120.0
C8—C9—H9	120.3	C55—C54—H54	120.0
N2—C10—C9	124.4 (3)	C54—C55—C56	120.2 (4)
N2—C10—H10	117.8	C54—C55—H55	119.9
C9—C10—H10	117.8	C56—C55—H55	119.9
C16—C11—C12	119.1 (3)	C55—C56—C51	120.5 (3)
C16—C11—P1	123.2 (3)	C55—C56—H56	119.8
C12—C11—P1	117.7 (3)	C51—C56—H56	119.8
C11—C12—C13	119.0 (4)	C66—C61—C62	118.5 (3)
C11—C12—H12	120.5	C66—C61—P2	118.3 (2)
C13—C12—H12	120.5	C62—C61—P2	123.1 (3)
C14—C13—C12	120.5 (4)	C61—C62—C63	120.1 (3)
C14—C13—H13	119.7	C61—C62—H62	120.0
C12—C13—H13	119.7	C63—C62—H62	120.0
C15—C14—C13	120.5 (4)	C64—C63—C62	120.6 (4)
C15—C14—H14	119.8	C64—C63—H63	119.7
C13—C14—H14	119.8	C62—C63—H63	119.7
C14—C15—C16	119.7 (4)	C65—C64—C63	119.6 (3)
C14—C15—H15	120.1	C65—C64—H64	120.2
C16—C15—H15	120.1	C63—C64—H64	120.2
C11—C16—C15	121.2 (4)	C64—C65—C66	121.0 (4)
C11—C16—H16	119.4	C64—C65—H65	119.5
C15—C16—H16	119.4	C66—C65—H65	119.5
C26—C21—C22	118.1 (3)	C65—C66—C61	120.3 (3)
C26—C21—P1	124.1 (3)	C65—C66—H66	119.9
C22—C21—P1	117.8 (3)	C61—C66—H66	119.9

Symmetry codes: (i) −*x*+1, −*y*+2, −*z*+1; (ii) −*x*−1, −*y*+1, −*z*+2.

### Hydrogen-bond geometry (Å, º)

**Table:**

*D*—H···*A*	*D*—H	H···*A*	*D*···*A*	*D*—H···*A*
C9—H9···Cl2^iii^	0.93	2.82	3.669 (4)	153

Symmetry code: (iii) *x*+1, *y*, *z*.
